# Supplementary material for: Model-based cost-effectiveness estimates of testing strategies for diagnosing hepatitis C virus infection in Central and Western Africa
Source: PLoS One. 2020 Aug 24;15(8):e0238035. doi: 10.1371/journal.pone.0238035 (PMC7446873; doi:10.1371/journal.pone.0238035)
Supplement: S1 Table — Abbreviations: Ab, antibody; cAg, core antigen; DBS, dried blood spot; lab, laboratory; POC, point of care; RNA, ribonucleic acid; S, strategy; Ven, Venepuncture. (DOCX) [file pone.0238035.s001.docx]

**S1 Table. Expected cost per screened individual and numbers of true positive, false positive, false negative, and true negative cases per 10,000 screened individuals under the base-case assumptions, excluding a seroprevalence value assumed at 46.9%.**

| **Strategy** | **Cost (€)** | **True positives** | **False positives** | **False negatives** | **True negatives** |
| --- | --- | --- | --- | --- | --- |
| S_12_: *POC HCV-RNA* | 14.28 | 3,136 | 128 | 148 | 6,589 |
| S_5_: *POC HCV-Ab 🡪 POC HCV-RNA* | 14.28 | 3,120 | 27 | 163 | 6,690 |
| S_8_: *POC HCV-Ab 🡪 Lab HCV-cAg (venepuncture)* | 23.65 | 3,051 | 17 | 232 | 6,700 |
| S_9_: *POC HCV-Ab 🡪 Lab HCV-cAg (DBS)* | 26.41 | 2,506 | 38 | 778 | 6,678 |
| S_10_: *Lab HCV-cAg (venepuncture)* | 33.69 | 3,051 | 17 | 232 | 6,699 |
| S_6_: *Lab HCV-Ab (venepuncture) 🡪 Lab HCV-cAg (venepuncture)* | 34.30 | 3,067 | 81 | 217 | 6,636 |
| S_11_: *Lab HCV-cAg (DBS)* | 39.14 | 2,452 | 38 | 831 | 6,679 |
| S_7_: *Lab HCV-Ab (DBS) 🡪 Lab HCV-cAg (DBS)* | 40.20 | 2,518 | 181 | 765 | 6,535 |
| S_3_: *POC HCV-Ab 🡪 Lab HCV-RNA (venepuncture)* | 40.30 | 3,264 | 4 | 20 | 6,712 |
| S_4_: *POC HCV-Ab 🡪 Lab HCV-RNA (DBS)* | 43.06 | 3,202 | 28 | 82 | 6,688 |
| S_ref_: *Lab HCV-Ab (venepuncture) 🡪 Lab HCV-RNA (venepuncture)* | 50.50 | 3,264 | 4 | 20 | 6,712 |
| S_2_: *Lab HCV-Ab (DBS) 🡪 Lab HCV-RNA (DBS)* | 55.48 | 3,134 | 28 | 150 | 6,689 |

Abbreviations: Ab, antibody; cAg, core antigen; DBS, dried blood spot; lab, laboratory; POC, point of care; RNA, ribonucleic acid; S, strategy; Ven, Venepuncture.
