# Supplementary material for: Model-based cost-effectiveness estimates of testing strategies for diagnosing hepatitis C virus infection in Central and Western Africa
Source: PLoS One. 2020 Aug 24;15(8):e0238035. doi: 10.1371/journal.pone.0238035 (PMC7446873; doi:10.1371/journal.pone.0238035)
Supplement: S2 Table — Abbreviations: Ab, antibody; DBS, dried blood spot; GDP, gross domestic product; HCV, hepatitis C virus; HE, health expenditure; lab, laboratory; POC, point of care; RNA, ribonucleic acid; S, strategy; WHO, World Health Organization. (DOCX) [file pone.0238035.s002.docx]

**S2 Table. Expected cost per screened individual and expected numbers of true positive, false positive, false negative, and true negative cases for achieving the WHO HCV testing targets by implementing the testing strategies S_5_ [*POC HCV-Ab 🡪 POC HCV-RNA*] and S_4_ [*POC HCV-Ab 🡪 Lab HCV-RNA (DBS)*] in 31.6% and 30.8%, respectively, of the general population of Cameroon, Côte d’Ivoire, and Senegal.**

| **Country and strategy** | **Cost /**  **screened individual (€)** | **Target of 30% of HCV-infected individuals diagnosed** | | | |  | **Target of 90% of HCV-infected individuals diagnosed** | | | |
| --- | --- | --- | --- | --- | --- | --- | --- | --- | --- | --- |
|  |  | **True positives** | **False positives** | **False negatives** | **True negatives** |  | **True positives** | **False positives** | **False negatives** | **True negatives** |
| **Cameroon** |  |  |  |  |  |  |  |  |  |  |
| S_5_: *POC HCV-Ab 🡪 POC HCV-RNA* | 8.32 | 146,015 | 1407 | 7,649 | 4,324,921 |  | 438,045 | 4,221 | 22,946 | 12,974,763 |
| S_4_: *POC HCV-Ab 🡪 Lab HCV-RNA (DBS)* | 12.73 | 146,015 | 1443 | 3,729 | 4,214,519 |  | 438,045 | 4,330 | 11,186 | 12,643,558 |
| **Côte d’Ivoire** |  |  |  |  |  |  |  |  |  |  |
| S_5_: *POC HCV-Ab 🡪 POC HCV-RNA* | 7.94 | 66,413 | 735 | 3,479 | 4,467,773 |  | 199,238 | 2,205 | 10,437 | 13,403,318 |
| S_4_: *POC HCV-Ab 🡪 Lab HCV-RNA (DBS)* | 8.83 | 66,413 | 754 | 1,696 | 4,353,799 |  | 199,238 | 2,262 | 5,088 | 13,061,396 |
| **Senegal** |  |  |  |  |  |  |  |  |  |  |
| S_5_: *POC HCV-Ab 🡪 POC HCV-RNA* | 7.77 | 19,593 | 278 | 1,026 | 2,924,721 |  | 58,779 | 834 | 3,079 | 8,774,163 |
| S_4_: *POC HCV-Ab 🡪 Lab HCV-RNA (DBS)* | 8.49 | 19,593 | 285 | 500 | 2,850,096 |  | 58,779 | 855 | 1,501 | 8,550,289 |

Abbreviations: Ab, antibody; DBS, dried blood spot; GDP, gross domestic product; HCV, hepatitis C virus; HE, health expenditure; lab, laboratory; POC, point of care; RNA, ribonucleic acid; S, strategy; WHO, World Health Organization.
