# Supplementary material for: Model-based cost-effectiveness estimates of testing strategies for diagnosing hepatitis C virus infection in Central and Western Africa
Source: PLoS One. 2020 Aug 24;15(8):e0238035. doi: 10.1371/journal.pone.0238035 (PMC7446873; doi:10.1371/journal.pone.0238035)
Supplement: S1 Text — (DOCX) [file pone.0238035.s003.docx]

**S1 Text. Sensitivity analysis: impact of variations in the ratio of the screening uptake rate of decentralized strategies to the screening uptake rate of centralized strategies.**

Below a threshold value of 2.4, the cost-effectiveness ranking of the strategies did not differ from that of the base-case. Above this threshold, S_6_ [*Lab HCV-Ab (venepuncture) 🡪 Lab HCV-cAg (venepuncture)*] became the least expensive strategy but presented a diagnostic accuracy much lower than the other un-dominated strategies, namely S_5_ [*POC HCV-Ab 🡪 POC HCV-RNA*] and S_3_ [*POC HCV-Ab 🡪 Lab HCV-RNA (venepuncture)*]. For instance, in a scenario considering a screening uptake rate of 80% and 30% for decentralized and centralized strategies, respectively (ratio = 2.7), S_5_[Ab_POC 🡪 RNA_POC] and S_3_ detected about three times more TP cases than S_6_ [*Lab HCV-Ab (venepuncture) 🡪 Lab HCV-cAg (venepuncture)*] (132 and 141 additional cases per 10,000 screened individuals for S_5_ [*POC HCV-Ab 🡪 POC HCV-RNA*] and S_3_ [*POC HCV-Ab 🡪 Lab HCV-RNA (venepuncture)*], respectively, as compared to the 76 cases detected by S_6_ [*Lab HCV-Ab (venepuncture) 🡪 Lab HCV-cAg (venepuncture)*], see Table A below). The corresponding ICER of S_5_ [*POC HCV-Ab 🡪 POC HCV-RNA*] to S_6_ [*Lab HCV-Ab (venepuncture) 🡪 Lab HCV-cAg (venepuncture)*] incurred an additional cost of €60.65 per additional TP case detected.

**Table A. Estimates of cost, effectiveness, and cost-effectiveness of testing strategies for detecting chronic hepatitis C cases under the hypothesis of an uptake rate of 30% for centralized strategies and of 80% for decentralized strategies.**

| **Strategy** | **Cost / screened individual**  **(€)** | **Number of true positive cases / 10,000 screened individuals** | **ICER**  **(€ / additional true positive cases detected)** | **Diagnostic accuracy**  **(%)**† | **Number of false positive cases / 10,000 screened individuals** | **Number of  false negative cases / 10,000 screened individuals** | **Number of true negative cases / 10,000 screened individuals** |
| --- | --- | --- | --- | --- | --- | --- | --- |
| S_6_: *Lab HCV-Ab (venepuncture) 🡪 Lab HCV-cAg (venepuncture)* | 5.75 | 76 |  | 29.93 | 1 | 6 | 2,917 |
| S_ref_: *Lab HCV-Ab (venepuncture) 🡪 Lab HCV-RNA (venepuncture)* | 6.27 | 81 | ‡ | 29.99 | 0 | 0 | 2,918 |
| S_5_: *POC HCV-Ab 🡪 POC HCV-RNA* | 6.55 | 208 | 60.65 | 79.87 | 2 | 11 | 7,780 |
| S_8_: *POC HCV-Ab 🡪 Lab HCV-cAg (venepuncture)* | 7.20 | 203 | ‡ | 79.83 | 1 | 15 | 7,780 |
| S_9_: *POC HCV-Ab 🡪 Lab HCV-cAg (DBS)* | 7.39 | 167 | ‡ | 79.45 | 3 | 52 | 7,779 |
| S_3_: *POC HCV-Ab 🡪 Lab HCV-RNA (venepuncture)* | 8.36 | 217 | 1,895.29 | 79.98 | 0 | 1 | 7,781 |
| S_4_: *POC HCV-Ab 🡪 Lab HCV-RNA (DBS)* | 8.55 | 213 | ‡ | 79.92 | 2 | 5 | 7,779 |
| S_10_: *Lab HCV-cAg (venepuncture)* | 10.29 | 76 | ‡ | 29.60 | 35 | 5 | 2,883 |
| S_12_: *POC HCV-RNA* | 11.42 | 209 | ‡ | 78.42 | 148 | 10 | 7,634 |
| S_7_: *Lab HCV-Ab (venepuncture) 🡪 Lab HCV-cAg (DBS)* | 19.87 | 163 | ‡ | 79.41 | 3 | 55 | 7,778 |
| S_2_: *Lab HCV-Ab (venepuncture) 🡪 Lab HCV-RNA (DBS)* | 21.06 | 208 | ‡ | 79.88 | 2 | 10 | 7,779 |
| S_11_: *Lab HCV-cAg (DBS)* | 32.16 | 168 | ‡ | 77.39 | 210 | 51 | 7,571 |

The model’s parameters, except for the uptake rates, were set at their base-case values.

Abbreviations: Ab, antibody; cAg, core antigen; DBS, dried blood spot; ICER, incremental cost-effectiveness ratio; lab, laboratory; POC, point of care; RNA, ribonucleic acid; S, strategy; Ven, Venepuncture.

†Diagnostic accuracy: sum of the true positive and true negative rates.

‡Dominated strategy.
