# Supplementary figures and images for: Model-based cost-effectiveness estimates of testing strategies for diagnosing hepatitis C virus infection in Central and Western Africa
Source: PLoS One. 2020 Aug 24;15(8):e0238035. doi: 10.1371/journal.pone.0238035 (PMC7446873; doi:10.1371/journal.pone.0238035)

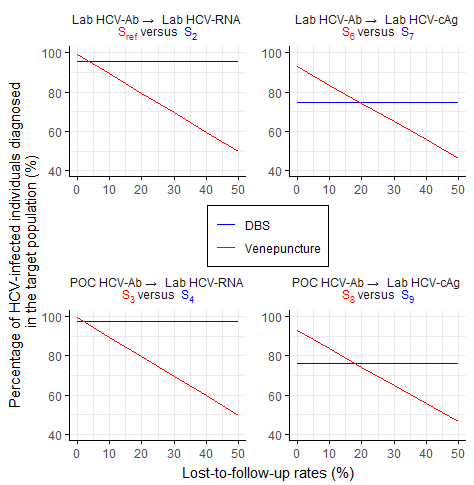

Supplement: S1 Fig — Each graph shows the results of the sensitivity analysis for strategies based on the same testing sequence, i.e., strategies including the same tests (same biomarker and same test setting: POC versus laboratory) and the same number of steps. The strategies of each pair differ only on the kind of samples used: venous blood samples (red line) or DBS (blue line). All of the model’s other parameters were set at their base-case values. Abbreviations: DBS, dried blood spot; lab, laboratory; POC, point of care; RNA, ribonucleic acid; S, strategy. (TIFF) [file pone.0238035.s004.tiff]

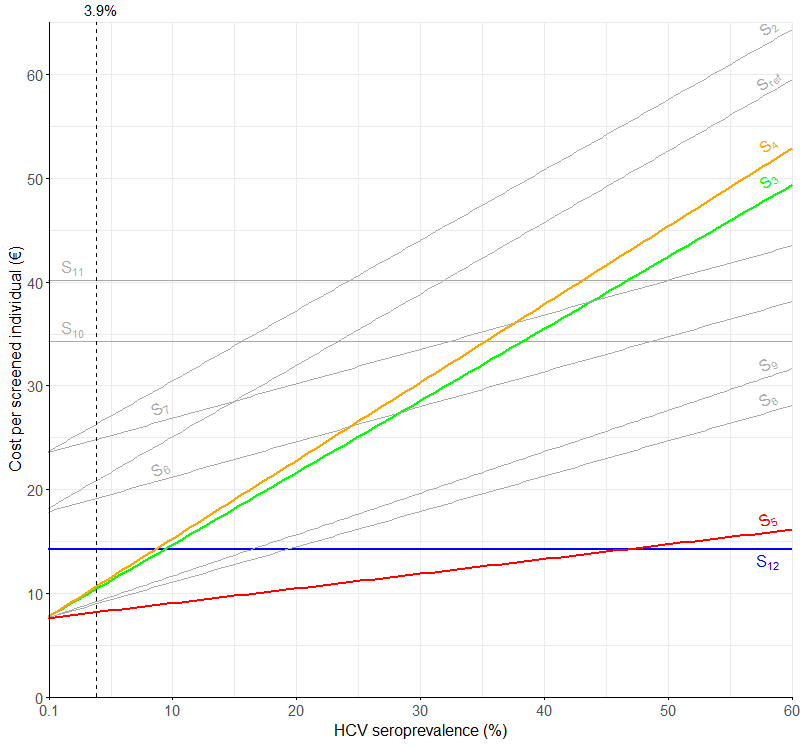

Supplement: S2 Fig — All of the model’s parameters, except HCV seroprevalence, were set at their base-case values. The dashed vertical line represents the base-case value for HCV seroprevalence (3.9%). The colored lines correspond to the strategies that were consistently dominant in the cost-effectiveness analysis. Abbreviations: DBS, dried blood spot; lab, laboratory; POC, point of care; RNA, ribonucleic acid; S, strategy. (TIF) [file pone.0238035.s005.tif]

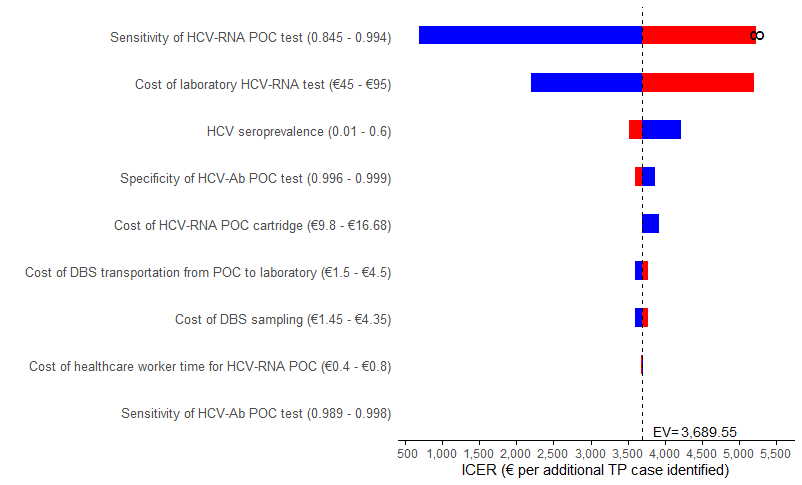

Supplement: S3 Fig — In each of the explored scenarios (top to bottom), a model parameter’s value was changed in comparison to the base-case. The vertical line reflects the ICER of S4 [POC HCV-Ab → Lab HCV-RNA (DBS)] versus S5 [POC HCV-Ab → POC HCV-RNA] under the base-case assumptions. The blue portion of a bar represents the parameter range from the low uncertainty value to the base-case, while the red portion represents the parameter range from the base-case to the high uncertainty value. For each of these scenarios, the figure indicates how the ICER changed in comparison to the base-case. For sensitivity values of an HCV-RNA POC greater than 0.98, S5 [POC HCV-Ab → POC HCV-RNA] became more effective than S4 [POC HCV-Ab → Lab HCV-RNA (DBS)], resulting in a negative ICER, designated here by the “∞” symbol. Abbreviations: Ab, antibody; DBS, dried blood spot; EV, expected value; HCV, hepatitis C virus; POC, point of care; RNA, ribonucleic acid; S, strategy. (TIF) [file pone.0238035.s006.tif]
