# Supplementary material for: Model-based cost-effectiveness estimates of testing strategies for diagnosing hepatitis C virus infection in Central and Western Africa
Source: PLoS One. 2020 Aug 24;15(8):e0238035. doi: 10.1371/journal.pone.0238035 (PMC7446873; doi:10.1371/journal.pone.0238035)
Supplement: Attachment — Submitted filename: Response to Reviewers.pdf [file pone.0238035.s007.pdf]

Response to Reviewers

Reviewer #1

Thank you for conducting this model and reporting using the CHEERS guidance, it certainly helps in the review process. Please consider the following comments to help you through the peer-review process:

1. Health Sector Only - The 1st and 2nd Panels on Cost Effectiveness both recommend health economists take a societal perspective in CEA with the 2nd panel recommending to present both a "health sector" and a "societal" amount. Can the authors comment further on why items such as patient time, travel, and productivity were not considered?

Re: We fully understand your concern. In order to address this point, in the method section, the initial subsection entitled "Outcomes" was renamed "Outcomes, Time Horizon, and Perspective" and the following paragraph was added at the end of this subsection (lines 158-168):

"A one-time screening based on a mass population approach was considered. The time horizon of all effectiveness outcomes investigated in this study was thus immediate (number of individuals screened, TPs, TNs…), and therefore no discounting rate was applied to health outcomes and associated costs. Such a time horizon questioned the adoption of a societal perspective in the cost-effectiveness analysis, especially when considering indirect costs: indeed, the relative short-term losses of work productivity between diagnosis strategies were likely uninformative while potential patient time and travel costs for diagnosis inherently constitute intangible costs that cannot be accurately estimated. Therefore, even if patient travel time and associated costs were indirectly considered since sensitivity analyses explored the impact of loss to follow-up, the perspective adopted in the present cost-effectiveness analysis should be considered as a perspective from the health sector."

2. Nomenclature - While the strategy names are logical and clear for a modeler, I'm worried the "average" reader will have a difficult time following the paper. Have you considered naming your strategies in a way that would be easier to read?

Re: The strategies' names have been changed throughout the text and in the tables (1, 4 and 5), Figure 2 and supporting information (Tables S1 and S2, S1 text, S1 Fig). For instance, S_4 is now referred to as S_4 [POC HCV-Ab → Lab HCV-RNA (DBS)] instead of S_4[Ab_POC→RNA_lab_DBS].

3. Smarter screening? - Physicians who determine an individual has a very low probability of disease (based on interview) can have a major impact on the effectiveness of screening. Applying a mass-population screening may be inappropriate for a variety of reasons. This variable would likely be the same for all 12 strategies, so it wouldn't impact the ICER, but can you comment on how your approach may bias the budget impact?

Re: We thank you for this thoughtful comment and accordingly, the following text was added in the Discussion section (lines 469-476):
"Finally, the combination of the following elements suggests that a more selective screening approach (risk-based, cohort-based) might be more appropriate than the mass population screening approach presented in the budget analysis (Table 5): the scarcity of resources, the low HCV seroprevalence in the general population of the study countries (1-4.9%), and the previous identification of risk factors for HCV infection in some countries (49-51). Nevertheless, Table 5 also

indicates that even a selective strategy targeting only 30% of the population would require a substantial increase of public health resources.”

Reviewer #2

The authors present a thought provoking and rigorous study comparing the cost-effectiveness of 12 different testing strategies for HCV screening in LMICs with the base case data for this study specifically from Cameroon, Cote d'Ivoire, and Senegal. The manuscript is presented in an intelligible way and I commend the authors for their thorough approach with a robust sensitivity analysis. It is almost ready to accept as is; however, I have a few minor suggestions that I think would improve the manuscript.

Re: We thank you for this general positive appraisal and your comments.

1. The abstract is very hard to read prior to having read the paper and I believe that the results should be re-written without using the testing abbreviations as these can be quite confusing. Rather please use language to describe the different testing scenarios and maybe only highlight a few in the abstract.

Re: We fully agree and accordingly, the abstract has been rephrased as suggested.

2. I would like to see uncertainty estimates presented in figure 2 and in figure S1 and S2. I believe that these would help further improve the transparency of an already fantastic analysis.

Re: In our view, uncertainty is already appropriately presented in these three figures for the following reasons.
The acceptability curves shown in Figure 2 were based on the replication of 10,000 simulations. Confidence intervals would therefore depend on the number of simulations, but the value of the information gained by changing this number would be little. For example, the 95% confidence interval of 10% with 10,000 and 100,000 simulations is around [9.5%-10.5%] and [9.8%-10.2%], respectively; therefore, multiplying by 10 the number of simulations shown in Figure 2 would not change the pattern presented at all. In addition, to our knowledge, all articles presenting acceptability curves are never presented with confidence intervals but show the expectations.
The situation is different for Figure S1 but drawing a confidence interval around the shown lines would also be inappropriate: the figure precisely presents the impact of the uncertainty of a parameter value (the proportion of lost to follow-up) on an outcome of interest (here, the expected percentage of HCV-infected individuals diagnosed in the target population). In other words, the percentage shown in ordinate is the true unbiased expected value issued from a simple analytical relationship of abscissa and ordinate, while in practice, the precision of this percentage will depend on the size of the target population chosen by the user (variable and unknown, one cannot draw "universal" confidence intervals that would make sense). In the end, the same rationale stands for Figure S2 which shows the true impact of varying seroprevalence (i.e. uncertainty on seroprevalence value) on the expected cost per screened individual (simple analytical and exact relationship between prevalence and cost).

According to this comment, in the ordinate legend of Fig.2, the initial text "percentage of simulations favouring …" was changed to "percentage of simulations (n = 10,000) favouring …" in order to recall to the reader that the pattern shown in the Figure is sufficiently precise and stable.

3. I was not able to find the CHEERS statement among the additional files if this could be made available as a supplementary file to the manuscript that would be great.

Re: We apologize for not having initially provided an additional file indicating where each item of the CHEERS checklist was addressed in our manuscript. The revised submission includes such a checklist for facilitating review process (pags 5-7 of the present document "Response to Reviewers"). However, this document cannot be supplied to the audience of PLoS ONE as a supplementary

material since the locations of each element will not match those of the final printed version of the manuscript.

4. If possible could all R code be made available for free either as supplementary files or through a link to a repository like GitHub or OSF.io

Re: According to your wish, we provide a link to a GiHub repository enabling the user to re-create all the figures of this submission. The following text was added in the revised version of the manuscript (Methods section, lines 255-257): "the corresponding R codes can be found at the following address: https://github.com/LeaDuchesne/Cost_effectiveness_HCV_testing_Central_Western_Africa".

CHEERS checklist when reporting economic evaluations of health interventions

Section/item	Item No	Recommendation	Reported on page No, line No
**Title and abstract**			
Title	1	Identify the study as an economic evaluation or use more specific terms such as "cost-effectiveness analysis", and describe the interventions compared.	Page 1
Abstract	2	Provide a structured summary of objectives, perspective, setting, methods (including study design and inputs), results (including base case and uncertainty analyses), and conclusions.	Pages 2-3, lines 1-33
**Introduction**			
Background and objectives	3	Provide an explicit statement of the broader context for the study.	Pages 5-6, lines 36-81
		Present the study question and its relevance for health policy or practice decisions.	Page 6, lines 83-95
**Methods**			
Target population and subgroups	4	Describe characteristics of the base case population and subgroups analysed, including why they were chosen.	Page 6, lines 93-97
Setting and location	5	State relevant aspects of the system(s) in which the decision(s) need(s) to be made.	Page 8, lines 121-128<br>Pages 23-25, lines 391-426
Study perspective	6	Describe the perspective of the study and relate this to the costs being evaluated.	Pages 9-10, lines 158-168
Comparators	7	Describe the interventions or strategies being compared and state why they were chosen.	Page 5, lines 68-70<br>Pages 6-7, lines 103-111<br>Table 1
Time horizon	8	State the time horizon(s) over which costs and consequences are being evaluated and say why appropriate.	Page 9, lines 158-161
Discount rate	9	Report the choice of discount rate(s) used for costs and outcomes and say why appropriate.	Page 12, lines 200-201
Choice of health outcomes	10	Describe what outcomes were used as the measure(s) of benefit in the evaluation and their relevance for the type of analysis performed.	Page 9, lines 154-57<br>Page 25, lines 438-445
Measurement of effectiveness	11a	*Single study-based estimates:* Describe fully the design features of the single effectiveness study and why the single study was a sufficient source of clinical effectiveness data.	NA

Section/item	Item No	Recommendation	Reported on page No, line No
	11b	*Synthesis-based estimates*: Describe fully the methods used for identification of included studies and synthesis of clinical effectiveness data.	Page 12, lines 187-190<br>Table 2<br>Table 3
Measurement and valuation of preference based outcomes	12	If applicable, describe the population and methods used to elicit preferences for outcomes.	NA
Estimating resources and costs	13a	*Single study-based economic evaluation:* Describe approaches used to estimate resource use associated with the alternative interventions. Describe primary or secondary research methods for valuing each resource item in terms of its unit cost. Describe any adjustments made to approximate to opportunity costs.	NA
	13b	*Model-based economic evaluation:* Describe approaches and data sources used to estimate resource use associated with model health states. Describe primary or secondary research methods for valuing each resource item in terms of its unit cost. Describe any adjustments made to approximate to opportunity costs.	Page 12, lines 193-199
Currency, price date, and conversion	14	Report the dates of the estimated resource quantities and unit costs. Describe methods for adjusting estimated unit costs to the year of reported costs if necessary. Describe methods for converting costs into a common currency base and the exchange rate.	Page 12, lines 198-200<br>Table 2<br>Table 3
Choice of model	15	Describe and give reasons for the specific type of decision-analytical model used. Providing a figure to show model structure is strongly recommended.	Page 8, lines 119-121
Assumptions	16	Describe all structural or other assumptions underpinning the decision-analytical model.	Pages 8-9, lines 121-139
Analytical methods	17	Describe all analytical methods supporting the evaluation. This could include methods for dealing with skewed, missing, or censored data; extrapolation methods; methods for pooling data; approaches to validate or make adjustments (such as half cycle corrections) to a model; and methods for handling population heterogeneity and uncertainty.	Pages 13-14, lines 203-256
**Results**			
Study parameters	18	Report the values, ranges, references, and, if used, probability distributions for all parameters. Report reasons or sources for distributions used	Table 2<br>Table 3

Section/item	Item No	Recommendation	Reported on page No, line No
		to represent uncertainty where appropriate. Providing a table to show the input values is strongly recommended.	
Incremental costs and outcomes	19	For each intervention, report mean values for the main categories of estimated costs and outcomes of interest, as well as mean differences between the comparator groups. If applicable, report incremental cost-effectiveness ratios.	Page 16, lines 261-269<br>Table 4
Table 4			
Characterising uncertainty	20a	*Single study-based economic evaluation:* Describe the effects of sampling uncertainty for the estimated incremental cost and incremental effectiveness parameters, together with the impact of methodological assumptions (such as discount rate, study perspective).	NA
	20b	*Model-based economic evaluation:* Describe the effects on the results of uncertainty for all input parameters, and uncertainty related to the structure of the model and assumptions.	Pages 18-20, lines 276-342<br>Figures 2, S1, S2 and S3<br>Table S1, S2 and S1 text
Characterising heterogeneity	21	If applicable, report differences in costs, outcomes, or cost-effectiveness that can be explained by variations between subgroups of patients with different baseline characteristics or other observed variability in effects that are not reducible by more information.	NA
**Discussion**			
Study findings, limitations, generalisability, and current knowledge	22	Summarise key study findings and describe how they support the conclusions reached. Discuss limitations and the generalisability of the findings and how the findings fit with current knowledge.	Pages 22-27, lines 369-486
**Other**			
Source of funding	23	Describe how the study was funded and the role of the funder in the identification, design, conduct, and reporting of the analysis. Describe other non-monetary sources of support.	Section "Financial disclosure" of the submission form
Conflicts of interest	24	Describe any potential for conflict of interest of study contributors in accordance with journal policy. In the absence of a journal policy, we recommend authors comply with International Committee of Medical Journal Editors recommendations.	Section "Competing interests" of the submission form
